# Implementing an Integrated Generalist-Led Inpatient Care Model: Results of a Mixed-Method Evaluation

**DOI:** 10.5334/ijic.6963

**Published:** 2023-09-21

**Authors:** Jennifer Sumner, Kimberly Teo, Cherylanne Tan, Sin Hui Neo, Lin Hui Lee, Brian Ng, Yee Wei Lim

**Affiliations:** 1 Medical Affairs – Research Innovation & Enterprise, Alexandra Hospital, National University Health System, Singapore; 2 National University of Singapore Institute of Systems Science, Smart Health Leadership Centre, Singapore; 3 Department of Medicine, Yong Loo Lin School of Medicine, National University of Singapore, Singapore

**Keywords:** hospital-based, chronic disease, health services research, implementation

## Abstract

**Introduction:**

Healthcare integration has become prevalent as health systems manage a growing population of older adults with multi-morbid conditions. The integrated general hospital (IGH) is the latest example of how services can be remodelled to achieve greater care integration.

**Methods:**

We conducted a mixed-method evaluation to identify factors impacting the implementation and effectiveness of the IGH model. Data were collected through in-depth interviews (n = 15) and focus group discussions (n = 8 groups) with hospital staff, and a staff survey (n = 226).

**Results:**

Staff perceived improvements in clinical practice and better clinical outcomes for patients. The care model empowered nursing and allied health staff through a more collegial team structure. However, staff reported an unequal workload distribution; a third reported burnout; and some observed inconsistencies between leaders’ aspirations for IGH and what was happening on the ground. For IGH to sustain, staff’s education on the IGH model needs to be improved. Further examination of work processes is recommended to boost staff morale and prevent burnout.

**Conclusion:**

Overall, IGH provided better integrated, team-based care. The model challenged traditional team structures and empowered staff to expand their roles and responsibilities. Policymakers could consider the IGH model a successful approach for integrating services across the care continuum.

## Introduction

Care integration is widely adopted to improve the quality of care and service efficiency [1,2]. Greater integration of services is important as health systems are faced with a growing number of older adults with multi-morbid conditions [3,4]. In Singapore, the proportion of citizens aged 60 years and above is projected to reach 40% by 2050 [5]. In response to this demographic trend, Singapore has attempted to integrate care in several forms in the past two decades. Most recently, there was a reconsideration of hospital-based care — the Integrated General Hospital (IGH) — which is the focus of this study.

Launched in 2018 at Alexandra Hospital, the IGH model’s objective is to shift hospital care from a specialist-dominant to a generalist-led model. The following principles guide IGH: First, care is not operationalised around specialisms; rather, patients are managed holistically under a generalist care team. In contrast to traditional inpatient care, which is organised around specialist services, IGH has no specialist wards. Occasionally, specialists are consulted, but this is the exception rather than the norm. Second, IGH provides acute and post-acute care on one site. In usual practice, patients who have recovered from an acute illness and require further rehabilitation would be sent to a separate community facility. IGH improves care continuity by removing inter-site transfers. Third, IGH care teams are guided by a “one patient, one care team” principle. Care teams proactively review patients’ care arrangements and consolidate care by reducing unnecessary specialist visits to other hospitals. Upon discharge, patients are also encouraged to seek follow-up care with a “principal physician” in IGH.

We conducted a mixed-method evaluation to identify factors that might have impacted the implementation and effectiveness of the IGH model. We focused on the staff’s perspectives of inpatient care, perceived effectiveness, scalability, and implementation challenges. The evaluation also captured views on the evolution of the model since its inception. The study objectives were:

1. To elicit staff’s perspectives of the IGH model and its implementation.
2. To determine the model’s effectiveness, examined through a qualitative lens.
3. To consider the scalability and replicability of the model.

## Methods

**Figure 1:**
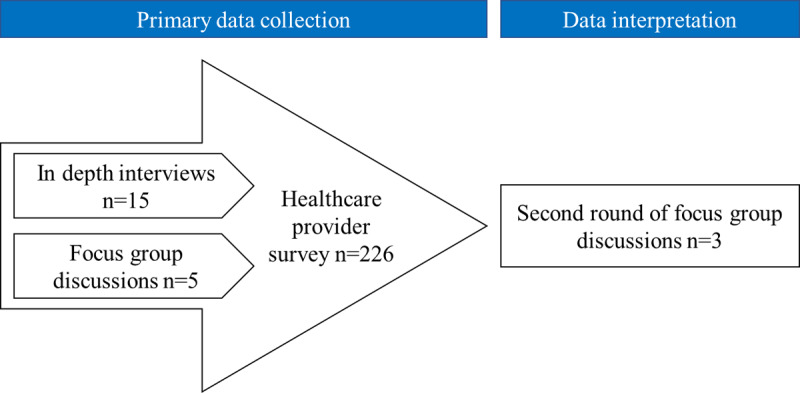
Mixed-method approach.

We applied a sequential, convergent, mixed-method study design (Figure 1). In the first phase, we conducted semi-structured interviews and focus group discussions to elicit views about the IGH model and its implementation. Interviews were conducted until data saturation was reached. After analysing data from the first phase, We designed and administered a staff survey. Lastly, we presented data from the interviews and the staff survey in a second round of focus group discussions (with participants who had not been interviewed previously). The second round of discussions explored the interpretation of the study findings and recommendations for the future.

We utilised the Comprehensive Framework for Implementation Research (CFIR) to guide our evaluation [6]. The CFIR comprises of five domains that might influence intervention implementation and effectiveness: intervention characteristics, internal setting, external setting, process of implementation, and characteristics and attitudes of the individuals involved in the intervention. The interview guides and staff survey were informed by the CFIR domains. The study is reported according to the Standards for Reporting Implementation Studies checklist [7]. The study was ethically reviewed and approved by the National Healthcare Group Domain Specific Review Board (2019/00308).

### Intervention

A generalist-led team-based care principle drives the IGH model. Patients remain on one site under the care of one team from start to end. In contrast to traditional inpatient care, IGH has no specialist wards. IGH is structured around five hospital-based programmes: Well (health prevention), FAST (inpatient care), Chronic (outpatient care), Healthy Ageing (inpatient and outpatient care), and Palliative Care. The team-based approach is facilitated through key IGH features outlined in Table 1.

**Table 1:** Description of the Integrated General-led Hospital (IGH) care model components.


IGH COMPONENTS	DEFINITION

A principal physician	A ‘principal’ physician is identified for every patient admitted to the acute inpatient wards. The principal physician is typically an internal medicine physician or a specialist acting as a generalist. The principal physician uses a holistic approach to ensure all the patients’ needs are addressed. Post-discharge, the principal physician continues to follow-up with the patient in the outpatient setting.

Acuity grading	Patients admitted to the inpatient wards are graded on a three-level acuity system: L1 (recovery and rehabilitation), L2 (sub-acute), and L3 (acute). The acuity grading system helps prioritise medical and nursing care. Acuity grading is evaluated daily to reflect patients’ care needs. L1 patients (i.e., those with mainly rehabilitative or social care needs) are typically managed by nurse clinicians, while physicians primarily manage L3 patients.

Multidisciplinary team meetings	The multidisciplinary team meetings are weekly meetings involving care team members. Any member of the care team may lead the meeting. The team develops a comprehensive care plan for patients, for inpatient and post-discharge care needs.

Care consolidation	Care consolidation seeks to improve care continuity and coordination by i) identifying a principal physician for a patient’s outpatient appointments, and by ii) reducing multiple specialist appointments. Eligibility is assessed upon admission (i.e., multiple morbidities and under the care of multiple specialists). If eligible, nurses introduce the service to patients and/or their families. After discussion, selected specialist appointments are cancelled, and the identified principal physician takes over. Although care is consolidated under a generalist physician, specialist advice is still sought when necessary.


### Participants

Hospital staff involved in inpatient care (at Alexandra Hospital) were invited to participate in a series of interviews (n = 15) or focus group discussions (n = 5 groups). Participants were recruited from different departments and job grades to gather a broad spectrum of perspectives (e.g., ground-up versus leadership views). Participants included physicians, nurses, pharmacists, medical social workers, human resources managers, therapists, and care managers.

For the survey, an electronic version or paper copies were made available to all staff. Participant details are reported in Table 2.

**Table 2:** Characteristics of staff survey respondents.


CHARACTERISTIC (n = 226)		

Sex, n (%):	Female	181 (80)

Role, n (%):	Allied Health professional	9 (4)

	Care Manager	4 (2)

	Medical Technologist	7 (3)

	Nurse	135 (60)

	Therapist – OT/PT/ST	13 (6)

	Pharmacist	19 (8)

	Physician	10 (4)

	Patient Service Associates (PSA)	12 (5)

	Social Worker	11 (5)

	Other	6 (3)

Experience, n (%):	<1 year	29 (13)

	1 year to <2 years	27 (12)

	2 years to <5 years	57 (25)

	5 years to < 10 years	30 (13)

	≥10 years	47 (21)

	Unknown	36 (16)

Employment status, n (%):	Full-time	217 (96)

	Full-time (appointed at ≥2 institutions)	6 (3)

	Part-time	3 (1)

Involved in inpatient care, n (%):	No	12 (5)

	Sometimes	16 (7)

	Yes	198 (88)


Results of the initial interviews, focus group discussions, and the staff survey were then presented in three further focus group discussions (in March 2021) – one with physicians, one with nurses, and an allied health group (total participants n = 16).

### Focus groups and interviews

A trained qualitative researcher (YWL) conducted the first round of interviews and focus group discussions between October 2019 – March 2020. Each focus group discussion involved three to six participants and was audio-recorded. Consent was taken, and recordings were transcribed verbatim by three research assistants (KT, SHN, CT).

### Staff survey

A staff survey was developed to further explore the issues identified from the interviews and focus group discussions. Topics covered by the survey included: Acuity transition, multidisciplinary team meetings, care consolidation, general perceptions of IGH, the opportunity for staff growth and professional development. The survey was launched between October–November 2020.

### Qualitative data analysis and synthesis

Qualitative data were collected and reported according to the COREQ checklist (Consolidated criteria for reporting qualitative research) [8]. We utilised the five dimensions from the CFIR framework to guide our data analysis [6]. In addition, a ‘suggestion’ theme emerged from the data and was included during coding. *Suggestion* refers to information on how the model could be improved. Data were mapped according to what IGH model component it relates to (e.g., acuity grading) and the corresponding CFIR domain. Coding and analysis were conducted in MS Office Word, Excel, and the Miro Whiteboard. KT and CT coded the data independently, and then the analysis was discussed as a group. Differences in opinion were mutually reconciled with YWL.

### Statistical analysis

Analyses were performed in STATA v15.0 (STATA Corp, College Station, Texas, USA). Summary statistics are presented as mean (with standard deviations, SD) or proportions. Incomplete responses were removed.

## Results

The results are presented in three sections: Overall effectiveness, implementation, and sustainability and scalability. We draw on data from the survey, focus group discussions, and interviews in each section. Additional qualitative data, which exemplifies the barriers, and enablers of IGH care implementation, are summarised in Appendix 1.

### Overall effectiveness of the IGH model

Staff recognised IGH as a distinct care model that combines traditional acute and rehabilitative care on one site. Respondents largely agreed (81%) that the IGH model improves patients’ outcomes.

IDI01: “Acute hospitals deal with acute problems and once the patient is deemed as stable but still requiring medical help, they will be transferred to a community-hospital*. And that’s where IGH model is sort of a hybrid between the two where you play both roles under one roof.”

[*community hospitals traditionally deliver rehabilitative care]

Senior physicians recognised that for effective care integration to occur, other care team members must actively participate in care planning. Staff frequently commented on the cultural shift, noting a flatter team structure, the approachability of physicians, and the greater voice and responsibility given to allied health colleagues. Over 40% of survey respondents agreed that the IGH model is less physician-centric and staff felt appreciated within the care team (82%).

*FGD01 (E): “It feels more collegial kind of working relationship.”*

The perceived improvements in clinical care were also facilitated by multidisciplinary team meetings. These meetings were highly valued by participants (78%). MDT meetings enabled staff to share information and align on care plans. Almost all survey respondents (98%) felt that multidisciplinary meetings lead to better discharge planning for patients and helped to improve the clinical care of patients (94%).

*FGD01 (F): “The multidisciplinary team meetings twice a week is a good platform where everyone can come together, discuss patients socially, psychosocially, rehab-wise, to make their inputs known, so we can plan for successful discharge.”*

Team-based care also increased the opportunities for interaction and communication, and over time, forged stronger collaboration between staff groups. Ninety-three percent of respondents felt that they were able to work effectively with their colleagues as a team.

*IDI02: “I believe, compared to when we started off, we were not confident because we – everybody all came from a hospital background where you don’t get to talk like that to a doctor. But here, I would say I can see that my team, after a year, the confidence is there because they (doctors) do hear (us), they see the doctors are listening.”*

The practice of team-based care was particularly evident when implementing the acuity grading system, where different care team members would step up and lead in care depending on patient’s acuity status.

*IDI05: “I think a lot of the discharge planning depends on the therapist, so they find they have more voice and their views are taken seriously.”*

Overall, the IGH model was perceived as effective, and staff felt it improved patient outcomes. Many of the care model components were perceived to facilitate improvements in clinical care.

### Implementation

#### Leadership

Some misalignments between the IGH vision and clinical practice were noted. Therapists (56%), especially, reported that the original IGH vision is different from existing practices. In certain instances, participants felt the direction of IGH was unclear. For example, there was a lack of direction on how community outreach activities should proceed.

*FGD01(F) “I don’t have a clear vision of where the hospital’s actually going, in terms of developing each domain or each specialty”*

*IDI02 “The community part of it (IGH) is, I have no idea where it’s going. I feel there’s a lack of leadership.”*

As the hospital scaled up, certain challenges were evident on the ground. When more wards were opened, teams on the ground were left struggling to cope due to limited manpower.

*FGD02 (B): “Recently they’ve [management] been opening new wards when we’ve just settled down in a ward, then they just cut our manpower into half. Then we have to put in more effort and more manpower and more hours to our work.”*

Development of IGH-related policies were also hindered by the lack of institutional autonomy. Often, health system-level policies influenced Alexandra Hospital’s policies.

*IDI08: “The policies lack clarity. They just take National University Hospital policies and Ng Teng Fong General Hospital policies, and when they can’t get anyone to agree, they will leave certain sections vague, right? Some of the policies I personally find were not drawn to our advantage because we are small”*

#### Work processes

Although IGH is intended to be a generalist-led care model, only 44% agreed that the IGH model is representative of generalist-led care. Interviewees questioned whether doctors can truly act as generalists for specialist cases, with 78% of survey respondents agreeing that specialists are still required. The limited availability of services and specialists at Alexandra Hospital (which would require transfer to other hospitals) remained a contentious point in follow-up focus group discussions.

*FGD02 (B): “It’s a good idea but maybe because our facilities are not really (big)enough, we still need to consult a lot of other specialists because our doctors are not really specialists in other care (needs).”*

The acuity grading system was felt to be used too subjectively, despite guidelines (72% agreed). Interviewees suggested a two-tier system could be simpler while others felt the middle tier (L2) was still useful for accommodating differing views. Of those surveyed, 63% agreed an L2 status was still useful.

*FGD01 (B): “I guess there’s no objectivity to it? It’s all ‘I may feel it’s L2, he may feel it’s L3’…I don’t even know if there is a better way to do it.”*

Staff also reported that workload, according to the acuity grading system, was not always well represented for each professional groups. For instance, the intensity of the workload for physiotherapists could be the same regardless of the patient’s acuity status. Therapists would assess and initiate therapy when patients were still acute (L3) and continue to manage patients to a less acute phase (L1). In contrast, the doctor’s workload was primarily in the acute phase of the patient’s stay in the hospital.

*IDI05: “People have always wanted to see how does rehab fit into this L3, L2, L1 and many times, they will say ‘Oh L3 the patient is not so well so they would require lower level of therapy, compared to L1…I want to clarify that it’s not always true.”*

*FGD09 (B): “Sometimes I think even though patients step down from L3 down to L2 or even L1, from the nurses’ perspective, workload may still be heavy. It depends on the activities of daily living needs.”*

Although multidisciplinary team meetings (MDTs) facilitated team-based care, they created stress amongst participants when different professional groups’ objectives were dissimilar. For example, physicians would like to discharge patients once medical issues have resolved, but physiotherapists want more time to rehabilitate patients before discharge.

*FGD07 (A): “I do feel the pressure that the team, the ward team is actually wanting this patient to be out as soon as possible.”*

In follow-up focus group discussions, participants expressed that the conduct of MDTs has improved in terms of a more focused agenda, better timings, and shorter duration of meetings. For instance, meetings were shortened by prioritising discussions on patients who needed multidisciplinary input, rather than all patients.

Interviewees were frequently conflicted about the role of care consolidation. Although many noted the benefit of reducing appointments from a patient’s perspective, some felt that specialist-led care for chronic conditions worked, and others doubted their own ability to act as a ‘generalist’.

*FGD03 (D): “Using the IGH model, I do see that the benefit of cutting down appointments for patients.”*

*FGD01 (F): “I think perhaps we’re sometimes being a bit too eager in consolidating…I mean, I’m pretty sure that the patient has been very well managed by the sub-specialties.”*

*FGD03 (B): “What if I get into trouble, is there any legal coverage for me because I’m not really trained in general medicine?”*

Follow-up interviewees felt the future of care consolidation depended on more staff involvement. Care managers were suggested as one group who could better facilitate care consolidation.

#### Managing Staff

The regular rotation of junior physicians was a substantial barrier to the success of IGH, particularly affecting team-based care. New staff joining the institution were unfamiliar with the IGH model, which created tension within the team. New staff needed time to learn new processes and adjust their mindset. There was increased pressure on existing staff to educate new colleagues while taking on additional workload. In the follow-up round of focus group discussions, interviewees suggested the need for better staff orientation. Despite these challenges, 80% of staff were confident that team conflicts were well handled and successfully resolved.

*FGD08 (E): “Because every six months (there is a) change of a new round of medical officers, we have to restart the IGH (education) – what is IGH again?”*

A third of those surveyed agreed that IGH caused burnout. Burnout was linked to insufficient staffing, a greater breadth of responsibilities, and the misalignment of the original IGH vision and how it is being practised. A third agreed the original IGH vision is different from how it operates on the ground; therapists particularly agreed on this point (56%).

*FGD03 (B): “There’s no redundancy of roles so far and because you need to be efficient and we aspire to have no redundancy, I think that spills over to stress that you would feel.”*

*FGD08 (E): “So when IGH was pitched to us, we can say how long we want to rehab the patient. FGD8 (A): yes, that’s what I thought as well, but then slowly it became different.”*

*FGD10 (B): “You know (considering) bed occupancy ratio and discharge, you can’t tell me to discharge quickly and consolidate (care) and keep the patient in.”*

Some specialists taking on generalist medicine felt they’ve grown as clinicians, but it affected their professional development as a specialist.

*FGD01 (B): After rounding …there are many things…now I feel confident in managing on my own. And even this holistic patient care……there’s a lot to learn, there are many things that, being in a specialty for three years, we have forgotten.”*

*FGD01 (B): “I mean, to be frank, I suspect if people had options of getting a job in 100% sub-specialty versus Alexandra Hospital, they would go for the 100% sub-specialty most of the time, they won’t come just because they believe in a generalist model.”*

#### Infrastructure/IT support

Participants commented that some infrastructural support was lacking, such as an information technology system that can facilitate care consolidation. Almost half of the staff involved in consolidating care agreed that without access to relevant patient information, identification of the patients’ existing appointments is challenging (48%), and a fifth found the process to be time-consuming. The main reason for the difficulty in identifying patients’ appointments is the lack of access to other healthcare institutions’ data systems.

### Scalability and sustainability

Despite the perceived success of the IGH model, there were uncertainties around its scalability and sustainability. Approximately a third reported the IGH model would not be sustainable as the hospital develops, suggesting that the generalist-led approach would dilute as specialist services expand. The increase in specialist care was also seen as an obstacle to scalability from a financial point of view, as specialist care is costlier.

*IDI04: “How does this work out when we move to the new site, when we build a 800-bedder hospital; will this model still work? when we add a lot more specialties and a lot more infrastructure, will we truly be cost-efficient?”*

Outside of the hospital, scalability may be affected by a patient preference for usual specialist care and healthcare providers’ and policymakers’ reliance on existing organisational arrangements. For example, patients are familiar with being transferred to a secondary site (i.e., a community hospital) for ongoing rehabilitative care and do not understand that IGH consolidates care needs on one site. Likewise, policymakers have not adjusted financing models to accommodate the IGH care approach. A few participants explained that easy access to subsidised specialist outpatient care (in hospitals) impedes expansion of generalist medicine.

*IDI03 “So the scalability of IGH is not just within how we practise in the hospital, it has to be something that the nation, the community is ready to take on as well. It may involve different ways of looking at how we measure success, how we measure hospital effectiveness.”*

*IDI02: “As long as policies don’t change, or we are not given the autonomy to, you know, test things or make a difference on that level, right, it’s- I don’t think you can scale it up.”*

Patient characteristics were another factor discussed in relation to scalability. Many noted that the hospital’s catchment area (which has older adults with complex needs) is highly appropriate for the IGH model, but this may not be replicated in other regions.

*FGD01 (C): “I think we’re also at an advantage for this kind of IGH model is probably because of our catchment area. Because in this area it’s a huge population of elderly so in terms of their care needs it’s definitely greater so if you were to in-build the rehab component, follow up everything properly, time, the community, it makes more sense.”*

Within the hospital context, the presence of burnout was identified as a barrier to scalability and sustainability. Staff also felt IGH does not reduce costs (46%) or use manpower more efficiently (43%), which could prevent the model being replicated elsewhere.

## Discussion

In 2018, Singapore launched its latest attempt at integrating healthcare – the IGH model [9]. To understand implementation-related challenges, we conducted a mixed-method evaluation. Overall, the care model was seen to be successful in its aim to provide better integrated, team-based care. Staff perceived an improvement in clinical outcomes. The care model also empowered nursing and allied health staff through a flatter, more collegial team structure. However, we found barriers to the new model: Staff reported unequal workload distribution; a third experienced burnout; and there was a perceived inconsistency between leaders’ aspirations for IGH and actual practice. Investment in IT infrastructure and a reconsideration of healthcare financing policies are also needed to better facilitate the IGH model.

As with many countries, Singapore faces a significant increase in chronic disease burden [10]. Patients with chronic disease require integrated services and more holistic care, a role typically assumed by primary care practitioners [2]. However, this is more complex in Singapore, where primary care is largely privatised with poor integration with the wider health system, and hospital-based specialist-led care dominates care delivery. In such environments, IGH is one way to enable care integration and help reduce the need for costly specialist-led care [11].

Prior to IGH, care team structures tended to be hierarchical. Physicians, who have traditionally led teams, had to transfer some responsibility to allied health and nursing colleagues to execute a team-based approach. Generally, we found IGH to be an effective model for flattening team structures, but changing mindsets did not occur consistently. This is not surprising when staff who joined IGH came from other hospitals, taking with them past work cultures. As several participants expressed in interviews, more could be done to orientate new staff at the outset. Identifying and recruiting staff who already believe in generalist care could prevent disappointment and maintain staff morale. Nevertheless, the transition to a flatter team structure was well received.

As allied health and nursing staff responsibilities diversify, physicians – particularly specialists – need to adapt to the generalist approach. No longer can chronic conditions be managed in isolation, rather, a holistic view needs to be taken to avoid fragmented and poorly coordinated care [12]. This does not imply specialist care is obsolete; instead, their expertise must be used more efficiently. In our study, and in a similar study the generalist approach led to some unease amongst treating physicians [13]. For instance, some specialist physicians acting as generalists were concerned about how this would impact their career development. There is a perception that the specialist career track offers higher socio-economic standing compared to the generalist track [14]. Therefore, IGH may be at odds with the desired career expectations of specialist doctors. Although our evaluation suggests new ways of operating can succeed, the right operationalisation of ‘generalist-led’ and ‘specialist-led’ care has yet to establish.

As healthcare workers take on new roles and team structures change, it is important to be mindful of burnout. When IGH was first implemented, we found that staff were burdened by new work processes while simultaneously delivering care to patients. For example, nurses have more responsibilities in IGH than they are previously used to. An initial lack of staff numbers further impacted the pressure to adapt to IGH, although this did resolve over time. These transient stressors may be expected with any new care model. Other more fundamental issues are harder to resolve, such as understanding the identity of IGH as a combined acute and rehabilitative hospital rather than just an acute care hospital. If barriers to IGH are not addressed, the original intentions of the model may dilute, and staff will burnout. More frequent dialogues between leaders and staff on the ground would be needed to ensure everyone is aware of the evolution of the IGH vision and allow staff to provide input on how best to translate the vision into realistic plans.

In addition to staff burnout, having appropriate infrastructure can also impact the success of a care model. We found care processes were inhibited by insufficient IT infrastructure, a common barrier in other studies [15]. Current IT systems impeded the ability to access information outside the hospital to complete tasks such as consolidating patients’ care arrangements. The lack of IT integration also hinders work with external community partners, who continue care post-discharge. Since our evaluation, a new electronic medical system (EPIC) has been deployed across the healthcare cluster, alleviating some IT-related integration issues. However, future IT infrastructure development is needed with the primary care sector to facilitate seamless care transitions.

Appropriate financing was another implementation challenge, common to other integrated care models in the Asia-Pacific region [16]. For example, in the current financing environment, acute care and subacute care are funded separately because these services are predominantly delivered in different facilities. In IGH, acute and subacute care are delivered on the same site, but existing financing models do not account for this. There is a need to explore a “blended” payment approach that accounts for the difference in acute care and subacute care delivery costs.

Overall, our evaluation identified several factors that impacted the implementation of IGH. First, to execute the various aspects of the IGH model with consistency, time for staff to be familiar with IGH-related tasks will be important; having too many changes could introduce confusion and create heterogeneity of practice. Frequent staff rotation disrupts the learning process as well. Second, there is a need to inculcate a generalist mindset among physicians, particularly specialists working in the IGH environment. In addition, as nurses and allied health staff take on more clinical responsibilities, structured training for IGH staff would help improve collaboration among IGH care team members, delineating new roles and responsibilities with greater clarity. Finally, financing the IGH model requires re-examination for sustainability. For example, IGH care combines acute and subacute care; should IGH be reimbursed as acute care or subacute care that a community hospital provides?

### Strengths and limitations

We conducted a comprehensive mixed-method evaluation of the IGH model. However, the study was impacted by the emergence of COVID-19. Health institutions were re-organised to care for COVID-19 patients. As such, the staff survey was delayed. Changes to the care model (e.g., the introduction of telemedicine) may have impacted the survey findings. In addition, although the staff survey was open to all professional groups, only a limited number of physicians took part, limiting the representativeness of their responses. Finally, we did not evaluate the cost-effectiveness of the care model. Future work is needed to understand whether the IGH model warrants investment from a cost-benefit perspective.

## Conclusions

Overall, IGH was perceived as an effective model for delivering generalist-led care and improved patient outcomes. The model challenged traditional team structures and empowered nursing and allied health in their practice, leading to the expansion of their roles and responsibilities. However, implementation challenges were not trivial. For the IGH model to scale and sustain, greater consideration of institutional and national-level obstacles is needed.

## Additional File

The additional file for this article can be found as follows:

10.5334/ijic.6963.s1 Appendix 1. Tables 3 and 4.
