# Supplementary material for: Implementing an Integrated Generalist-Led Inpatient Care Model: Results of a Mixed-Method Evaluation
Source: Int J Integr Care. 2023 Sep 21;23(3):13. doi: 10.5334/ijic.6963 (PMC10516141; doi:10.5334/ijic.6963)
Supplement: Appendix 1. — Tables 3 and 4. [file ijic-23-3-6963-s1.pdf]

Appendix 1

Table 3 Examples of implementation barriers, by CFIR domain

CFIR domain	IGH related barrier	Example quotes
**Intervention characteristics**	Misalignment of IGH vision and clinical practice	IDI2: "I think we were pretty clear that IGH means everything happens here. But because at the end of the day, people are still being pushed for the length of stay, so I think the measures are totally wrong, if you ask me."
**Inner setting**	Staff rotation	FGD8 (E): "Maybe because the doctors every 6 months, we change a new round of MOs, then we have to restart the IGH – what is IGH again?"
	Misalignment of IGH vision and clinical practice	IDI1: "If the vision is not clear then everybody will be stubborn in their own way, then the MDT is a complete waste of time."
**Outer setting**	Lacking external partners	FGD1 (B): "The non-NUHS institutions is very difficult. You don't know the doctors and you don't know what they're being treated, you can't even see the notes. Within NUHS, it's easier, you just look at C-DOC, 'Oh stabilised, TCU six months, then okay, I can speak to cardio." [Care consolidation]
	Misalignment of IGH vision and clinical practice	FGD4 (D): "When family members have a misconception that we are like a community hospital. So many times they will request to extend their stay, and we will explain to them that when patient does not have a rehab potential, we can't allow them to stay longer than it's necessary."
**Individual characteristics (attitude)**	Cultural change required	IDI3: "This [IGH] requires a change in culture, which actually is a bit difficult considering that we have different teams that change, we even have doctors who rotate across different hospitals, so for them to accept different ways of working, sometimes it's little difficult."
**Implementation process**	IT infrastructure lacking	IDI6: "So every time when there is a change in acuity, the doctors have to pass us a form, I mean, they have to update us, for the ward clerks, they have to manually go into the system to update it, so it is a manual process to update it." [IT systems]

Table 4 Examples of implementation enablers, by CFIR domain

CFIR domain	IGH related enabler	Example quote
**Intervention characteristics**	Flatter team structure	IDI5: “The doctors, the consultants, the MOs, everyone are much more- much more approachable and they do take your views seriously, they don’t brush you aside.”
**Inner setting**	Supportive staff	FGD1 (E): “If we need help from other specialties in terms of referrals, I feel like it’s easy- it’s quite easy to call someone and ask for help and ask for advice here as compared to NUH.” [Another hospital in the cluster not using the IGH Model]
**Outer setting**	Belief in care consolidation	FGD3 (C): “If I’m in the shoes of an 80-year-old and I have at least four, five chronic conditions and see four, five different physicians, I would be very reluctant to go for my follow-ups, compared to coming to just one person” [Care consolidation]
**Individual characteristics (attitude)**	Empowering culture	IDI5: “At least the feel I have is very much, very much our views are more respected here, compared to back in NUH where we play more of a supporting role.” [Allied health perspective]
**Implementation process**	Holistic team approach<br><br>Increasing responsibilities of nurses and allied health	FGD4 (A): “Patient don’t have to move around, different transport, different therapists.”<br><br>IDI11: “I think it’s a good idea …now we have a whole workforce who is better equipped to provide care.” [Delegation of duties to support nurse-led care]
